# Topical Steroid and Fairness Cream Abuse in Facial Dermatoses: A Cross-Sectional Study at a Tertiary Care Center in Western Uttar Pradesh

**DOI:** 10.7759/cureus.104892

**Published:** 2026-03-09

**Authors:** Pihu Sethi, Kriti Maheshwari, Ekta Arora, Jasmeet Dhilon, Mehak Gupta

**Affiliations:** 1 Dermatology, Venereology and Leprosy, Government Institute of Medical Sciences, Greater Noida, IND; 2 Dermatology, Venereology and Leprosy, Yashoda Medicity, Ghaziabad, IND; 3 Pharmacology, Government Institute of Medical Sciences, Greater Noida, IND; 4 Medicine and Surgery, Government Institute of Medical Sciences, Greater Noida, IND

**Keywords:** fairness creams, india, steroid abuse, topical corticosteroids, tsdf

## Abstract

Background: Abuse of topical corticosteroids (TCS) and “fairness creams” on the face has become a significant dermatological problem in India, leading to a spectrum of adverse effects known as topical steroid-damaged/dependent face (TSDF). This study aims to assess the demographic profile of patients with facial TCS misuse, evaluate their perceptions and awareness regarding steroid use, and document the clinical adverse effects associated with misuse.

Methods: A prospective, questionnaire-based, observational cross-sectional study was conducted over two months (July-August 2025) in the Department of Dermatology, Government Institute of Medical Sciences (GIMS), Western Uttar Pradesh. Ninety-six consecutive patients with a clinical diagnosis of TSDF were enrolled. Data regarding demographics, indications for use, source of recommendation, type and potency of steroids, duration of use, and cutaneous adverse effects were recorded in a preformed questionnaire and analyzed using IBM SPSS Statistics for Windows, Version 28.0 (Released 2021; IBM Corp., Armonk, NY, USA).

Results: Out of the 96 patients, 66 (68.8%) were females, and 30 (31.3%) were males, with a mean age of 29.42 years. Rural residents constituted 51 (53.1%) participants. The majority of the patients belonged to lower socioeconomic groups (59.4%). Non-prescription use was reported by 62 (64.6%) patients, most commonly recommended by pharmacists (32, 51.6%). Clobetasol propionate was the most frequently used steroid by 43 (44.8%) patients, and 45 (46.9%) patients had used other superpotent corticosteroids. The mean duration of use of topical agents was 13.65 months. Common indications for usage included melasma (39, 40.6%), tinea (29, 30.2%), and acne (24, 25%). The most frequent adverse effects were hypopigmentation (84, 87.5%), cutaneous atrophy (84, 87.5%), hypertrichosis (69, 71.9%), acneiform eruptions (61, 63.5%), and telangiectasia (61, 63.5%). A large proportion of patients (92, 95.8%) were unaware of the contents of the creams, and 94 participants (97.9%) did not read product leaflets. The overall total economic burden was INR 1,72,659 (USD 1885), with marked individual financial expenditure.

Conclusion: Facial misuse of TCS is highly prevalent in Western Uttar Pradesh, largely driven by over-the-counter (OTC) availability, pharmacist recommendation, prescription by non-registered practitioners, and poor awareness. There is an urgent need for stricter regulation of potent steroids, banning irrational fixed-dose combinations, and large-scale public and pharmacist education to prevent further morbidity.

## Introduction

Topical corticosteroids (TCS), first introduced by Sulzberger and Witten in 1952 [1], have since become indispensable in dermatological practice because of their anti-inflammatory, immunosuppressive, vasoconstrictive, and antiproliferative properties [2]. When used judiciously, they play a vital role in the management of chronic inflammatory dermatoses, such as eczema, psoriasis, vitiligo, and lichen planus [2]. However, misuse of these agents has emerged as a major dermatological concern in India [3,4].

Unregulated over-the-counter (OTC) availability, aggressive marketing of irrational fixed-dose combinations (FDCs), and sociocultural ideals equating fair skin with beauty have fueled their rampant misuse [3-5]. Potent TCS, often marketed as fairness or anti-pigmentation creams, are frequently used without a medical prescription for conditions such as melasma, acne, freckles, and generalized hyperpigmentation [4,6]. As a result, cases of topical steroid-damaged/dependent face (TSDF), first described by Lahiri and Coondoo [7] and characterized by pharmacodependence, rebound erythema, telangiectasia, acneiform eruptions, hypertrichosis, dyspigmentation, and dermal atrophy, are on the rise [8-11].

This problem is particularly prevalent in rural areas, where the availability of dermatologists is limited (approximately 1 per 130,000 population), and awareness of the long-term adverse effects of topical steroids is poor [12,13]. Surveys have reported facial TCS misuse rates of up to 90% among unsupervised users [5,9,10].

Despite this growing burden, data from Western Uttar Pradesh remain sparse. Therefore, the present study was undertaken to (a) assess the magnitude of the problem with respect to sociodemographic factors, (b) recognize the relationship between steroid abuse and patients’ perceptions of cutaneous disease, (c) assess the level of awareness and attitudes toward the use of topical steroids and fairness creams, and (d) determine the spectrum of adverse effects associated with topical steroid misuse.

## Materials and methods

A cross-sectional observational study was conducted in the Department of Dermatology, Government Institute of Medical Sciences (GIMS), Western Uttar Pradesh, over a period of two months (July-August 2025). All patients with a history of TCS application on the face, either without prescription by a dermatologist or for a duration longer than prescribed, were screened and registered for further evaluation.

Patients presenting with cutaneous adverse effects attributable to TCS use as the chief complaint, with a history of regular use for ≥1 month or intermittent use for ≥3 months, were included in the study. Patients with clinical features suggestive of TCS abuse who were unable to recall or provide details regarding the topical preparation used, despite repeated probing, were excluded.

Patients fulfilling the study criteria underwent a detailed clinical evaluation, including comprehensive history-taking and thorough cutaneous examination. Sociodemographic data, such as age, sex, place of residence, educational status, and annual income, were recorded using a preformed questionnaire (Appendix 1). Information regarding TCS use, including the type of corticosteroid, duration of application, indication for use, source of recommendation or prescription, number of topical preparations used, and approximate cost of medication, was documented. Patients were also queried about their awareness of potential adverse effects, whether they had read the product information leaflet, and whether they were aware of the constituents of the topical preparation used. All cutaneous adverse effects attributable to TCS misuse were systematically recorded.

The study protocol was reviewed and approved by the GIMS Institutional Ethics Committee (Approval No. GIMS/IEC/HR/2025/68) prior to commencement. Written informed consent was obtained from all participants, and strict confidentiality of patient information was maintained throughout the study.

Data were entered into Microsoft Excel (Microsoft Corp., Redmond, WA, USA) and analyzed using IBM SPSS Statistics for Windows, Version 28.0 (Released 2021; IBM Corp., Armonk, NY, USA). Descriptive statistics were employed for data analysis. Categorical variables were expressed as frequencies and percentages, while continuous variables were expressed as means and ranges.

## Results

A total of 96 patients with a history of topical steroid application on the face were evaluated. Of them, 30 (31.3%) were male, and 66 (68.8%) were female, demonstrating a clear female preponderance. Among the participants, the most common occupation was housewife, reported in 37 (38.5%) cases. The age of the patients ranged from 13 to 56 years, with a mean age of 29.42 years. Of the 96 patients, 51 (53.1%) were residents of rural areas and 45 (46.9%) were from urban areas, highlighting the widespread nature of misuse. Educational attainment was heterogeneous: 24 (25%) patients had completed higher secondary education, 21 (21.9%) were graduates, 19 (19.8%) were illiterate, and 18 (18.8%) had completed secondary education. The majority of patients belonged to lower-income groups, with 57 (59.4%) classified in category 1 and 26 (27.1%) in category 2 (Table 1).

**Table 1:** Demographic characteristics of the patients (N = 96)

Demographic characteristics	Number (N)	Percentage (%)
Gender		
Male	30	31.3
Female	66	68.8
Resident-wise distribution of patients		
Rural	51	53.1
Urban	45	46.9
Educational level		
Illiterate	19	19.8
Lower primary (4th class)	1	1.0
Middle (8th class)	10	10.4
Secondary (10th class)	18	18.8
Higher secondary (12th class)	24	25.0
Graduate	21	21.9
Post-graduate	3	3.1
Socioeconomic status of the patients		
Below 1 lac	57	59.4
1-1.5 lac	26	27.1
1.5-2.5 lac	6	6.3
2.5-5 lac	6	6.3
Above 5 lac	1	1.0

Among the 96 patients assessed, 62 (64.6%) reported using topical steroids without a prescription, while 34 (35.4%) used them under medical prescription. Of the 34 prescribed users, the majority (23, 67.6%) had received prescriptions from unqualified practitioners (quacks), followed by general physicians (9, 26.5%), and dermatologists (2, 5.88%). Among the 62 patients who used topical steroids without a prescription, 32 (33.3%) obtained them directly from pharmacists, 17 (17.7%) were influenced by friends, 9 (9.37%) by relatives, 3 (3.1%) by nursing staff, and 1 (1.0%) patient reported media influence as the source of recommendation (Table 2).

**Table 2:** Source of prescription on topical steroid use (N = 96)

	Frequency	Percentage
Quacks	23	67.6
General physicians	9	26.5
Dermatologist	2	5.88
Pharmacists	32	33.3
Friends	17	17.7
Relatives	9	9.37
Nursing staff	3	3.1
Media	1	1.0

Steroid usage patterns

Clobetasol propionate was the most commonly used topical steroid, reported by 43 patients (44.8%), followed by betamethasone valerate 0.1% (36, 37.5%), mometasone furoate (14, 14.6%), and betamethasone dipropionate (3, 3.1%). The duration of steroid use ranged from a minimum of 1 month to a maximum of 120 months, with a mean duration of 13.65 months. Regarding potency, of the 96 patients, 45 (46.9%) had used Class I (superpotent) corticosteroids, 36 (37.5%) had used Class II (potent), and 14 (14.6%) had used Class IV (moderately potent) corticosteroids. The classification of corticosteroid potency was based on standard pharmacological criteria as described by Hengge et al. [12] (Table 3).

**Table 3:** Commonly used topical steroids by the patients (N = 96) and their indications

	Frequency	Percentage
Clobetasol propionate	43	44.8
Betamethasone valerate 0.1%	36	37.5
Mometasone furoate	14	14.6
Betamethasone dipropionate	3	3.1
Class I (superpotent)	45	46.9
Class II (potent)	36	37.5
Class IV (moderately potent)	14	14.6
Melasma	39	40.6
Tinea	29	30.2
Acne	24	25.0
Not known	4	4.2

Indications for use

The most common indication for the use of TCS among the 96 participants was melasma (39, 40.6%), followed by tinea (29, 30.2%) and acne (24, 25%) (Table 3).

Adverse effects

**Figure 1:**
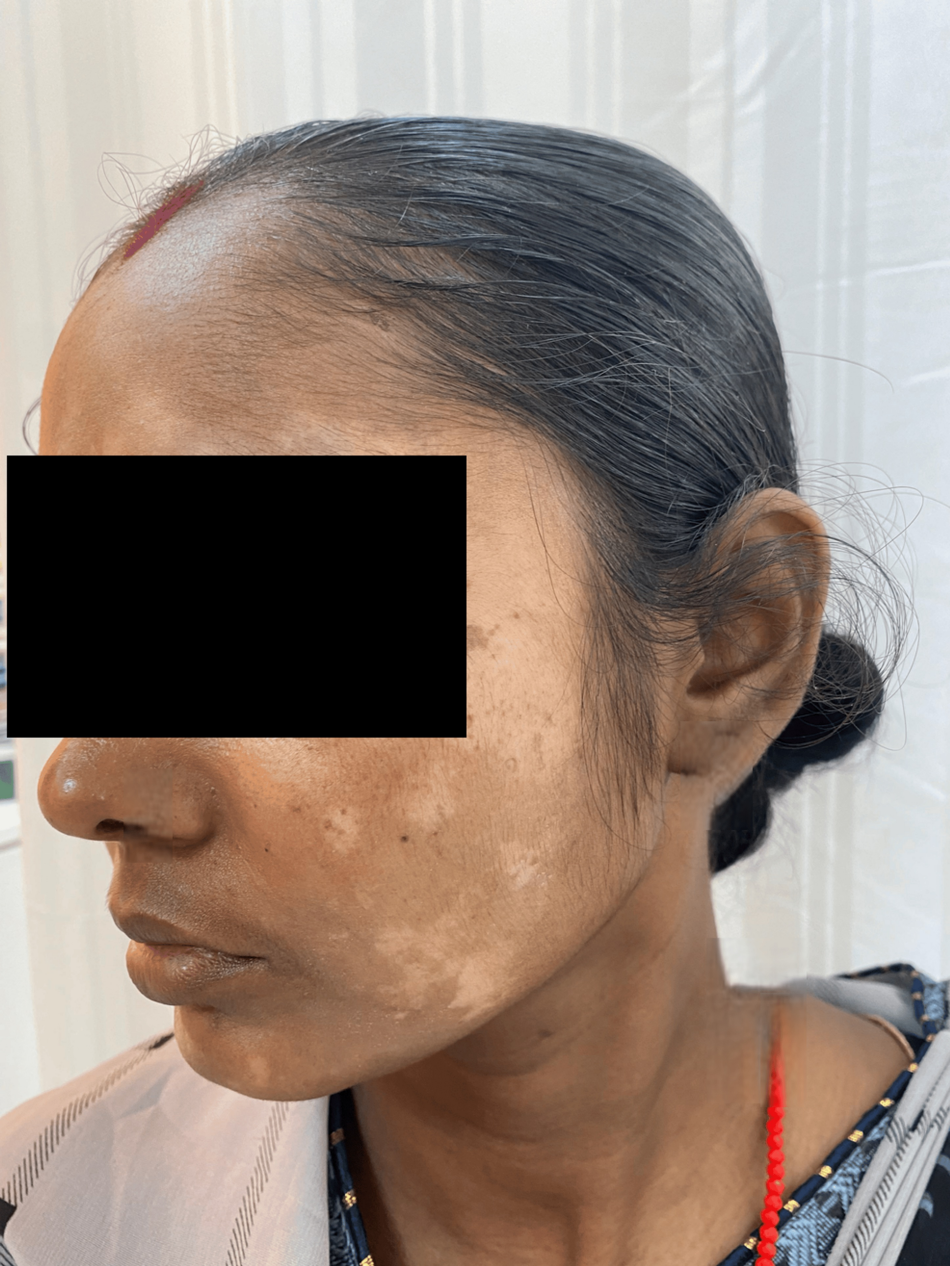
Hypopigmented macules and patches over the face

**Figure 2:**
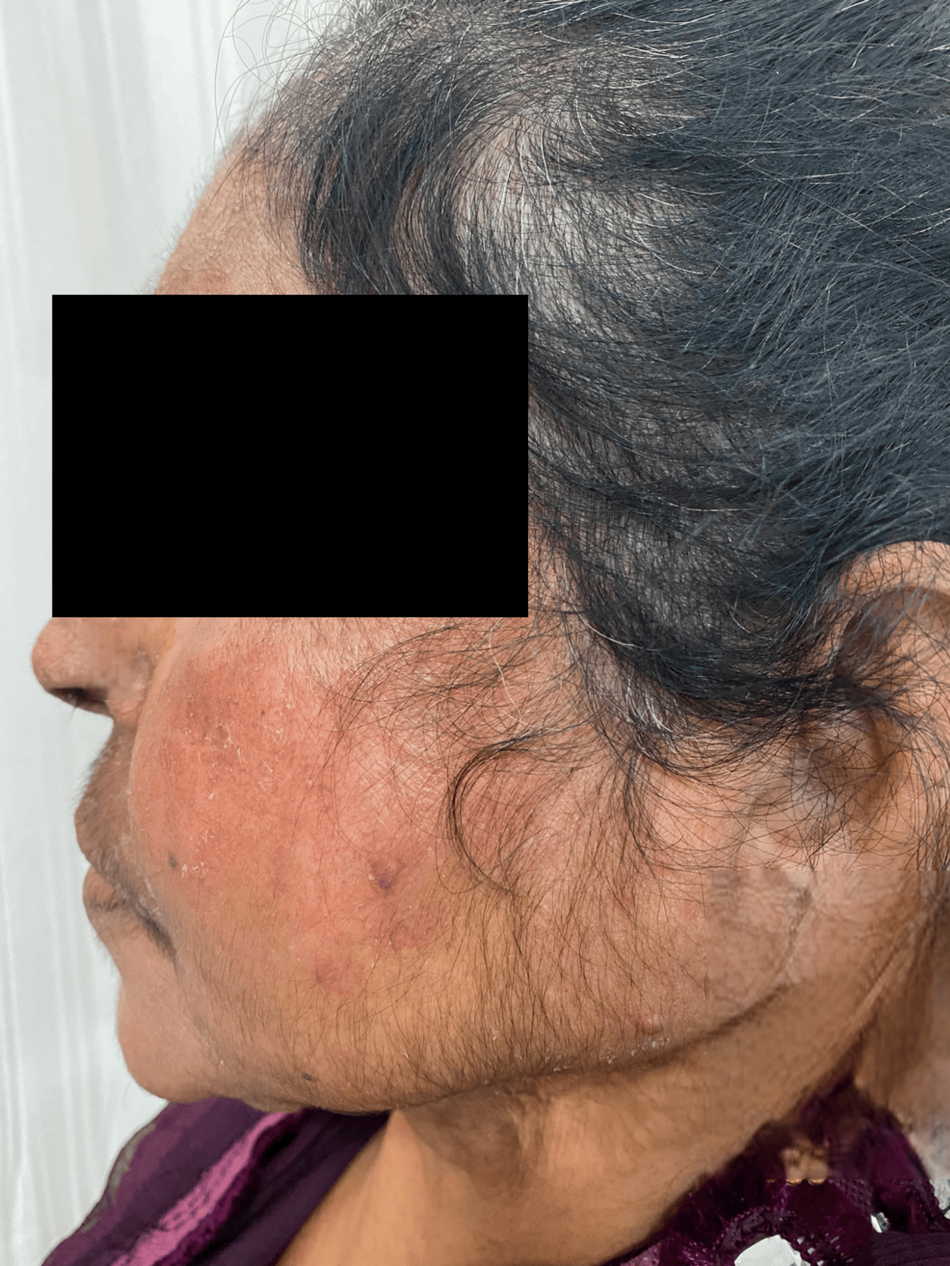
Hypertrichosis

**Figure 3:**
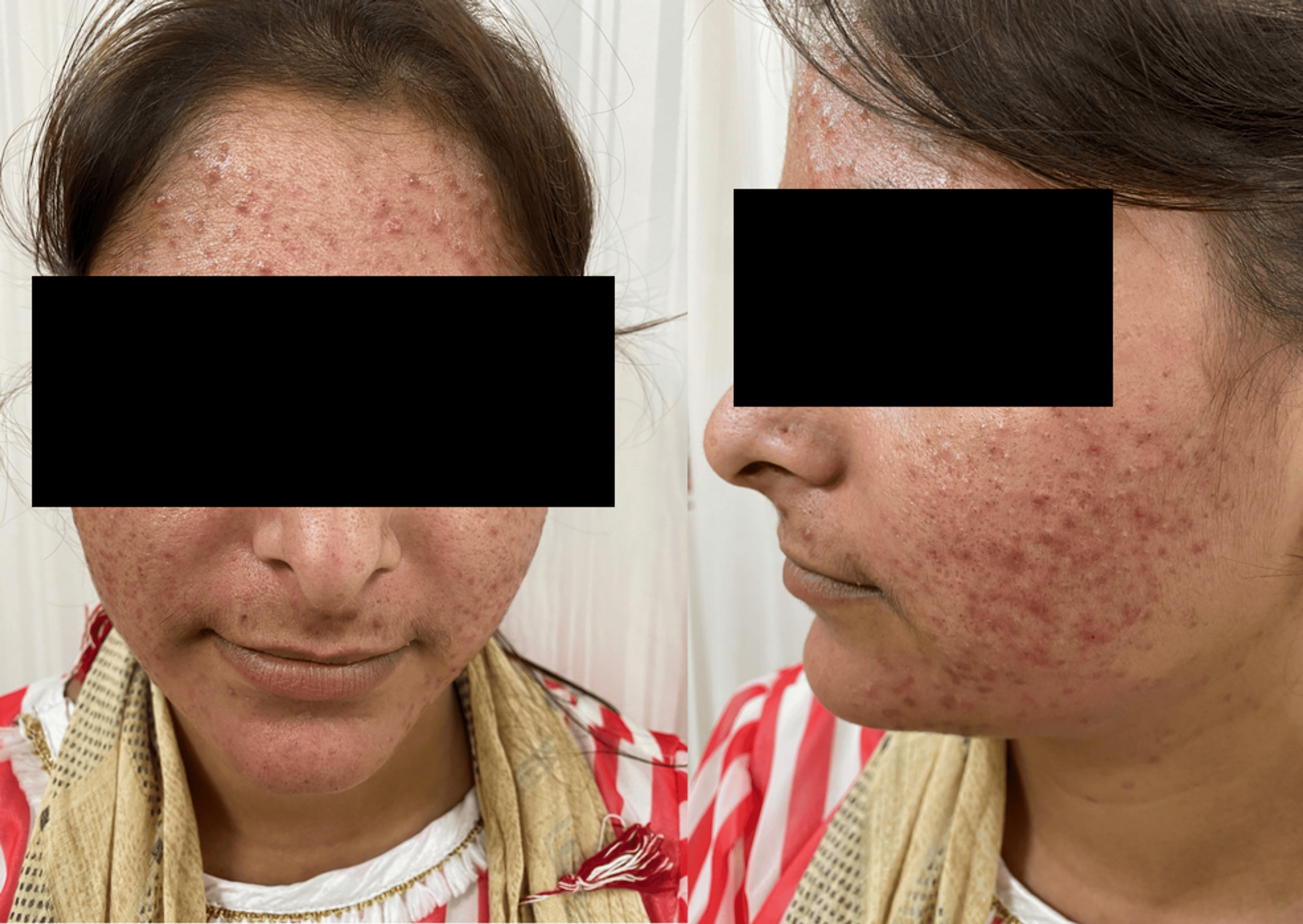
Acneiform eruptions

On cutaneous examination, the most frequent clinical findings were hypopigmentation, observed in 84 (87.5%) patients (Figure 1), and cutaneous atrophy, also noted in 84 (87.5%) patients. Hypertrichosis (Figure 2) was seen in 69 (71.9%) patients, while acneiform eruptions and telangiectasia (Figure 3) were each observed in 61 (63.5%) cases. Less common findings included erythema (6, 6.3%) and hyperpigmentation (2, 2.1%). All clinical findings, along with their respective frequencies and percentages, are detailed in Table 4.

**Table 4:** Cutaneous adverse effects observed in the patients (N = 96)

Clinical findings	Frequency	Percentage
Hypopigmentation	84	87.5
Cutaneous atrophy	84	87.5
Hypertrichosis	69	71.9
Acneiform eruptions	61	63.5
Telangiectasia	61	63.5
Erythema	6	6.3
Hyperpigmentation	2	2.1

Among the 96 patients included in the study, 92 (95.8%) were unaware of the constituents of the topical preparation used, while only 4 (4.2%) demonstrated knowledge of its contents. A total of 94 patients (97.9%) reported that they had not read the product leaflet or instructions, whereas only 2 (2.1%) had done so. Most patients (89, 92.7%) reported having received counseling regarding potential side effects from a doctor or pharmacist prior to use. The cumulative number of tubes used was 1,731, with the highest usage recorded in a single patient being 240 tubes, followed by 215 and 180 tubes. The total financial burden incurred by all patients in the study was ₹1,72,659. The highest expenditure by a single patient was ₹45,000 over a period of six months. Each tube costs approximately ₹250, and the patient used around 180 tubes during this duration.

## Discussion

This study demonstrates that misuse of TCS on the face remains a major dermatological problem in India. Similar to earlier reports, women and young adults constituted the majority of affected individuals in our study, consistent with findings by Saraswat et al. [14] and Coondoo [3]. The slightly higher proportion of rural patients (51, 53.1%) also aligns with observations by Thomas et al. [5], who reported that individuals in rural areas often have limited access to dermatologists and are more likely to obtain creams directly from pharmacies. The mean age of patients in our study (29.4 years) is comparable to that reported by Saraswat et al. [14], further emphasizing that young adults are particularly vulnerable. The predominance of rural patients suggests that limited specialist availability and weaker regulation of OTC creams may contribute to increased misuse in these settings [3,15].

A key finding of our study was that 62 (64.6%) patients were using topical steroids without any prescription. This pattern has been consistently reported in previous Indian studies by Nagesh and Akhilesh [4] and Rathi [6], who identified OTC availability as a major driver of misuse. Even among the 34 patients who reported prescription use, the majority of prescriptions (23, 67.6%) were issued by unregistered practitioners (quacks). Similar observations were reported in a community-based study from North India, where non-qualified practitioners played a significant role in steroid misuse [5].

Socioeconomic factors also appeared to contribute substantially. A large proportion of patients belonged to lower-income categories (Categories 1 and 2). These individuals are more likely to purchase inexpensive OTC fairness or combination creams from pharmacies rather than seek consultation with a dermatologist, thereby increasing the risk of steroid misuse. This trend has been similarly described by Coondoo [3] and others, who highlighted cost-related self-medication as a major contributor to abuse.

Among patients who used steroids without a prescription, pharmacists were the most common source of recommendation, accounting for 32 (33.3%) of the 62 non-prescription users. This finding is consistent with the study by Ashique et al. [13], who reported that pharmacy personnel often recommend potent topical steroids without adequate knowledge or counseling. Advice from friends, relatives, beauticians, and even nursing staff was also reported, a pattern observed in other South Asian studies [10,16].

A significant concern highlighted by this study was the lack of patient awareness. Of the 96 patients, 92 (95.8%) were unaware of the ingredients in the creams they used, and 94 (97.9%) had never read the product leaflet. One major contributing factor is the misleading marketing of fixed-dose combination (FDC) creams as fairness or antifungal products, which encourages inappropriate use [17,18]. Aggressive advertising and sociocultural pressure for “fairer” skin further promote unrealistic expectations and indiscriminate use. In our study, melasma was the most common self-diagnosed indication for topical steroid use, differing from some rural North Indian studies where tinea predominates [5].

Clobetasol propionate was the most commonly misused steroid, reported by 43 (44.8%) patients, consistent with findings from multiple studies across India and even exceeding rates reported in some regional surveys [17,19]. This underscores the urgent need for stricter regulatory measures, such as prominent warning labels or reclassification of potent topical steroids as prescription-only drugs. Many of these products are marketed as cosmetic creams in combination with other agents, leading to inappropriate use, delayed correct diagnosis, and complications such as steroid-modified fungal infections (tinea incognito) [17,18].

The easy availability of potent steroids without prescription has resulted in prolonged use, as reflected by the cumulative usage of 1,731 tubes in our study, with some patients using more than 200 tubes. Such long-term and repeated use is consistent with the concept of topical steroid dependence described by Lahiri and Coondoo [7] and Kligman and Frosch [8].

The most common adverse effects observed, such as hypopigmentation, cutaneous atrophy, hypertrichosis, acneiform eruptions, and telangiectasia, are well-documented consequences of prolonged topical steroid use and have been reported in earlier studies by Hengge et al. [12], Shrestha et al. [16], and Dey [19]. More severe adverse effects, such as striae or secondary infections, were less frequent in our study, likely due to the relatively shorter mean duration of misuse (13.65 months) compared to studies involving chronic long-term users [14].

Study limitations

The primary limitation of this study is its short duration of only two months. Larger studies with longer durations and increased sample sizes are required to better understand the magnitude of the problem. Additionally, community-based field surveys, in addition to hospital-based studies, would provide deeper insights into the true prevalence and patterns of misuse.

## Conclusions

Combination creams containing corticosteroids, hydroquinone, tretinoin, or antifungal agents are frequently used as a panacea for various dermatoses by general physicians and unregistered practitioners in the Indian setting. These medications are widely sold OTC without regulation and are misused by patients as routine cosmetic creams, often without any awareness of their long-term adverse effects. Overall, this study highlights the urgent need for stricter regulation of topical steroid availability, banning irrational FDCs, improving awareness among pharmacists and the general public, and educating patients about the risks associated with improper facial steroid use.

## Appendices

Appendix 1

**Table 5:** Questionnaire

S. no.		
1.	Name	
2.	Occupation	
3.	Age/sex	
4.	Place	
5.	Urban/rural	
6.	Literacy	
7.	Yearly income	Below 1 lac
		1-1.5 lac
		1.5-2.5 lac
		2.5-5 lac
		Above 5 lac
8.	Corticosteroid use/brand name	
9.	Potency	
10.	Duration of use	
11.	Indication	
12.	Prescription, non-prescription	Dermatologist/general physician/quack pharmacist/nursing staff/friends/relatives/media/advertisement/others
13.	Did you know its constituents	Steroids/hydroquinone
14.	Did you read the leaflet/usage instructions	
15.	Were you enlightened regarding the side effects by the doctor/pharmacists before	
16.	How many tubes were used	
17.	What is the price of each tube	
18.	Adverse effects	Tinea incognito/acne/atrophy/telangiectasia/hypopigmentation/striae/ICD/pyoderma/hypertrichosis/others
